# Preparation of Uniform PEG-PLLA Microspheres via Membrane Emulsification for Soft Tissue Filling Applications

**DOI:** 10.3390/jfb17020071

**Published:** 2026-01-30

**Authors:** Siqi Zhang, Yuan Gao, Danyang Wang, Yongjie Chi, Fang Wu, Lianyan Wang, Hailan Jin

**Affiliations:** 1 Key Laboratory of Bio-Based Material Science & Technology, Ministry of Education, Northeast Forestry University, Harbin 150040, China; zhangsiqi20010823@163.com; 2 Institute of Process Engineering, Chinese Academy of Sciences, Beijing 100190, China; gaoyuan@ipe.ac.cn (Y.G.); wangdanyang24@ipe.ac.cn (D.W.); chiyongjie21@ipe.ac.cn (Y.C.); shengh398@gmail.com (F.W.); 3 School of Chemical Engineering, University of Chinese Academy of Sciences, Beijing 100190, China; 4 College of Pharmacy, Heilongjiang University of Chinese Medicine, Harbin 150040, China

**Keywords:** membrane emulsification technology, PEG-PLLA microspheres, collagen regeneration, soft tissue filling

## Abstract

Skin aging could lead to dermal collagen loss and elastic fiber degradation, ultimately manifesting as skin laxity. We aimed to counteract this by using poly-L-lactic acid (PLLA) microsphere (MS)-based fillers to facilitate long-term volume restoration through collagen regeneration. However, conventional MSs exhibit limitations such as broad size distribution and surface irregularities, which are frequently associated with significant adverse reactions. This study employed shirasu porous glass (SPG) membrane emulsification to fabricate uniform and well-shaped polyethylene glycol-block-poly (L-lactic acid) (PEG-PLLA) MSs. A single-factor experiment was employed to optimize the parameters. The optimal preparation conditions for PEG-PLLA MSs were as follows: PEG-PLLA concentration of 40 mg/mL, polyvinyl alcohol (PVA) concentration of 0.5%, and magnetic stirring speed of 200 rpm. Under the optimal conditions, the average particle size of PEG-PLLA MSs was 58.982 μm, and the span value (SPAN) was 1.367. In addition, a cytotoxicity assay was performed, and the results revealed no significant toxicity of the MSs toward L929 mouse fibroblasts at concentrations below 500 μg/mL. Furthermore, PEG-PLLA MSs significantly enhanced the production of key extracellular matrix (ECM) components—type I collagen (Col-I), type III collagen (Col-III), and hyaluronic acid (HA)—while simultaneously alleviating cellular oxidative stress responses. This work offers a reliable and reproducible fabrication strategy for developing biocompatible MS fillers with controllable particle sizes.

## 1. Introduction

Skin aging is a complex biological process influenced by a combination of genetic regulation, metabolic dysregulation, environmental exposures (such as photoaging), and lifestyle factors [1,2]. With advancing age, the dermis undergoes substantial alterations, characterized by a progressive decline in collagen content, diminished skin elasticity, and fragmentation of elastic fibers. These changes disrupt the structural integrity of the elastic network, ultimately contributing to the formation of wrinkles [3].

Plastic surgery currently offers a range of established approaches for skin tissue reconstruction in the context of aging, including allograft implantation, autologous tissue grafting, autologous fat transplantation, and the use of injectable filler materials [4,5,6,7]. As minimally invasive techniques have advanced, injectable dermal fillers made from poly-L-lactic acid (PLLA) have become the preferred choice in plastic and reconstructive surgery. These fillers are favored for their high safety, minimal invasiveness, low infection risk, short recovery time, long-lasting results, and their ability to naturally improve skin aging [8,9].

Statistical analysis of adverse effects following the injection of the first-generation PLLA dermal filler revealed significant issues due to its irregular particle structure and overly sharp edges, which tends to trigger strong inflammatory reactions upon injection [10]. Additionally, most commercially available PLLA dermal fillers are characterized by microspheres (MSs) with non-uniform sizes and broad size distribution. However, achieving uniform particle size in MSs is essential for batch reproducibility and consistent therapeutic efficacy. The underlying rationale involves the size-dependent phagocytosis by macrophages. MSs exceeding 20 μm in diameter evade macrophage uptake, which prevents subsequent acute inflammation and minimizes injection-related adverse effects [11,12,13,14]. To further address the problem, shirasu porous glass (SPG) membrane emulsification technology was selected to prepare MS fillers with smooth surfaces, good morphology, and uniform controllable particle size. Nonetheless, the inherent hydrophobicity of pure PLLA can result in particle aggregation upon injection, increasing the risk of inflammatory nodule formation. The introduction of polyethylene glycol (PEG) blocks modulates the hydrophilic–lipophilic balance of PLLA, thereby markedly enhancing its biocompatibility and dispersion stability [15].

This study aims to establish an optimal membrane emulsification process for preparing polyethylene glycol-poly (L-lactic acid) (PEG-PLLA) MSs with a uniform size distribution (target: 30–60 μm). We hypothesize that these PEG-PLLA MSs will show good biocompatibility. Moreover, we further propose that they can stimulate fibroblasts to secrete key extracellular matrix (ECM) components, including the type I collagen (Col-I), type III collagen (Col-III), and hyaluronic acid (HA) and reduce cellular oxidative stress. These hypotheses will be systematically tested to provide a foundation for developing novel soft-tissue filler materials.

## 2. Materials and Methods

### 2.1. Materials and Reagents

The copolymer PEG-PLLA was provided by Imeik Technology Development Co., Ltd. (Beijing, China). Dichloromethane (DCM) and Polyvinyl alcohol (PVA) were purchased from Aladdin Biochemical Technology Co., Ltd. (Shanghai, China). Roswell park memorial institute 1640 medium (RPMI 1640), fetal bovine serum (FBS), penicillin–streptomycin solution (PS) and phosphate-buffered saline (PBS) were bought from Procell Life Science & Technology Co., Ltd. (Wuhan, China). Cell Counting Kit-8 (CCK-8) was obtained from Dojindo Laboratories (Kumamoto, Kyushu, Japan). The enzyme-linked immunosorbent assay (ELISA) kit was purchased from Dakewe Biotech Co., Ltd. (Beijing, China). Assay kits for malondialdehyde (MDA), catalase (CAT), and superoxide dismutase (SOD) were purchased from Adsbio Biological Technology Co., Ltd. (Nanjing, China). All other chemical reagents were of analytical grade.

### 2.2. Preparation of PEG-PLLA MSs

**Figure 1:**
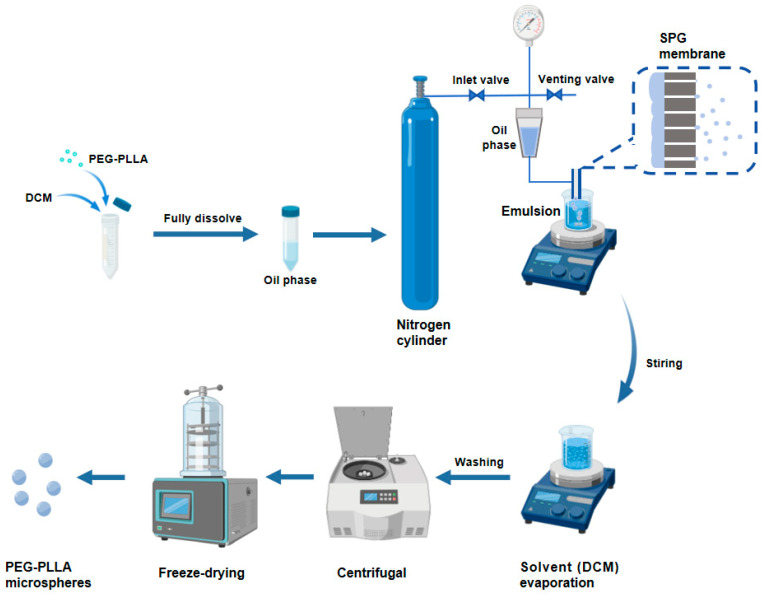
The preparation process of the PEG-PLLA MSs.

The PEG-PLLA MSs were fabricated by membrane emulsification. As shown in Figure 1, we accurately weighed 0.4 g of PEG-PLLA and dissolved it in DCM to form the oil phase. Subsequently, the oil phase was injected into the PVA solution through an SPG membrane tube with a uniform pore size of 15 μm using the membrane emulsification device, obtaining a PEG-PLLA MS suspension. The MS suspension was magnetically stirred at room temperature for 12 h to accelerate dichloromethane removal. Subsequently, the solidified MSs were collected and washed, followed by lyophilization to obtain the PEG-PLLA MS powder. 

### 2.3. Characterization of PEG-PLLA MSs

Scanning electron microscopy (SEM) (JSM-6700F, Jeol, Tokyo, Japan) was utilized to examine the morphology of MSs. A 200 μL aliquot of the MS suspension was applied onto a clean aluminum foil surface. After the samples were fully dried, they were sputter-coated with a gold-palladium alloy (30 mA, 120 s) and scanned at an accelerating voltage of 5.0 kV. Micrographs were acquired at magnifications of ×60 and ×300.

The particle size of the PEG-PLLA MSs was determined with a Laser Particle Size Analyzer (Mastersizer 3000, Malvern Instruments, Worcestershire, UK). The particle size distribution was evaluated by average diameter and SPAN and calculated from the volume distribution using the Mie model. The SPAN calculation formula is as follows:

$$\mathrm{SPAN}\;=\;\frac{D_{90}-D_{10}}{D_{50}}$$

where *D*_90_ is the particle size cumulative distribution at 90% by volume, *D*_10_ is at 10%, and *D*_50_ is at 50%.

The SPAN is a measure of how wide the size distribution or the flatness of the distribution curve. For SPAN ≤ 5, the size distribution is considered to be narrow. The measurements were performed 3 times for each sample.

### 2.4. Single-Factor Experimental Design to Optimize the Preparation Process

A single-factor experimental design was used to determine the initial range values of the following three extraction factors: PEG-PLLA concentration (20–50 mg/mL), PVA concentration (0.25–1%), and magnetic stirring speed (50–250 rpm). The dependent variable was the physicochemical properties of PEG-PLLA MSs, including morphology and particle size distribution.

### 2.5. Cell Culture and Viability Analysis

L929 cells, a fibroblast cell line derived from mouse subcutaneous connective tissue, were sourced from the National Experimental Cell Resource Sharing Platform (Beijing, China). Ethical approval was not required for this study, as it involved only commercially available cell lines and no human participants or animal subjects. All experiments were conducted in accordance with relevant guidelines and regulations. The cells were cultured in RPMI 1640 containing 10% FBS and 1% PS at 37 °C in a humidified atmosphere with 5% carbon dioxide. When cells reached 80% confluence, they were detached with 0.25% trypsin-ethylenediaminetetraacetic acid (EDTA) solution, resuspended in RPMI, and then seeded in a 96-well plate at a density of 5 × 10^3^ cells per well. After 24 h of incubation, various concentrations of samples (62.5, 125, 250, 500, 1000, 2000 μg/mL), which had been sterilized by gamma irradiation at a dose of 25 kGy, were added to the corresponding wells and incubated for an additional 24 h, 48 h, and 72 h. At each time point, 10 μL of CCK-8 solution was added to each well and incubated at 37 °C for 2 h. The absorbance was measured at 450 nm with a Varioskan LUX microplate reader (Thermo Fisher Scientific Inc., Waltham, MA, USA) and cell viability was assessed.

$$\mathrm{Cell}\;\mathrm{viability}\;(\%)=\frac{A-C}{B-C}\;\times \;100\%$$

In the formula, *A* represents the OD value of the sample group cells; *B* denotes the OD value of the control group cells; *C* corresponds to the OD value of the blank control.

### 2.6. Optimization of H_2_O_2_ Concentration

Following the cell culture and plating procedures outlined in Section 2.5, cells were cultured for an additional 24 h. Various concentrations of H_2_O_2_ (25, 50, 100, 200, 400, 600, 800, and 1000 μM) were then added to the designated wells and incubated for 24 h. Subsequently, 10 μL of CCK-8 solution was added to each well, followed by incubation at 37 °C for 2 h. Absorbance at 450 nm was measured using a Varioskan LUX microplate reader to determine cell viability in each group.

### 2.7. Evaluation of Col-I and Col-III Secretion Levels in Cells

**Figure 2:**
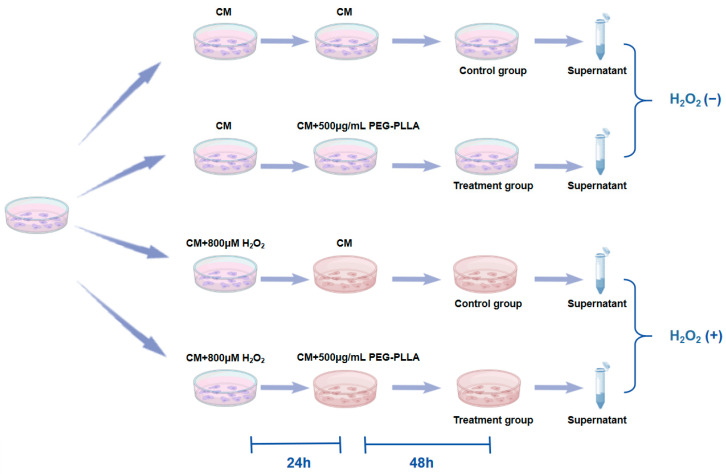
Experimental design for H_2_O_2_-induced fibroblast senescence and PEG-PLLA MS treatment.

To assess the secretion of Col-I and Col-III, an ELISA was performed. The grouping of cells and treatment regimens were shown in Figure 2. L929 fibroblasts were categorized into two groups: normal cells and oxidative stress-treated cells. Normal cells (normal group) were obtained by culturing normal L929 fibroblasts in complete medium (CM) for 24 h. To induce cellular oxidative stress, L929 cells were treated with CM containing 800 μM H_2_O_2_ for 24 h (oxidatively stressed group). Subsequently, both normal and oxidatively stressed cells were incubated with PEG-PLLA MS suspension (500 μg/mL) for 48 h. Control groups for both cell types were cultured in CM without MSs for the same period. After treatment, culture supernatants were collected and centrifuged at 15,000× *g* for 5 min. The concentrations of Col-I and Col-III in the supernatant were then quantified using a commercial ELISA kit. Absorbance was measured at 450 nm with a Varioskan LUX microplate reader.

### 2.8. Evaluation of Hyaluronic Acid (HA) Secretion Levels in Cells

The secretion of HA was assessed using an ELISA. Cell treatment and grouping followed the same protocol as described in Section 2.7. After treatment, the culture supernatant was collected and centrifuged at 15,000× *g* for 5 min. The concentration of HA in the supernatant was then quantified using a commercial ELISA kit. Finally, absorbance was measured at 450 nm with a Varioskan LUX microplate reader.

### 2.9. Evaluation of Oxidative Stress-Related Indicators in Cells

Oxidative stress was assessed by measuring the content of MDA and the activities of the antioxidant enzymes CAT and SOD cell modeling, and the experimental grouping followed the same protocol as described in Section 2.7. Cells from each group were collected, washed twice with PBS, and lysed by sonication. Subsequently, the MDA content and the activities of SOD and CAT in the lysates were quantified using specific commercial assay kits, following the manufacturers’ instructions.

### 2.10. Statistical Analysis

Data were presented as the mean ± standard deviation (SD). The normality of all data sets were assessed using the Shapiro–Wilk test. For comparisons between two groups that passed the normality test, Student’s t-test was applied; otherwise, the Mann–Whitney U test was used. For comparisons among multiple groups, data were analyzed by one-way analysis of variance (ANOVA) followed by Tukey’s post hoc test using GraphPad Prism version 10.1.2. The analysis yielded a *p*-value of less than 0.05, indicating statistical significance.

## 3. Results

### 3.1. Optimization of the PEG-PLLA MS Preparation Process

#### 3.1.1. Concentration of PEG-PLLA

**Figure 3:**
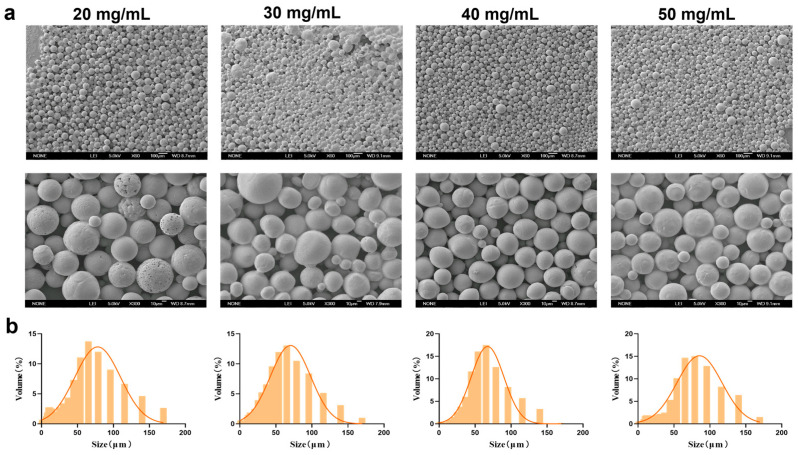
Characterization of MSs for PEG-PLLA concentration optimization. (**a**) SEM micrographs of PEG-PLLA MSs and (**b**) Particle size distribution of PEG-PLLA MSs.

Under fixed conditions of 0.5% PVA and a stirring speed of 150 rpm, PEG-PLLA MSs were prepared at concentrations of 20, 30, 40, and 50 mg/mL. The morphology and particle size distribution of the MSs were evaluated using SEM and laser diffraction analysis, respectively. As shown in Figure 3, the proportion of MSs in the target size range of 30–60 μm was 45%, 51%, 52%, and 42% for the 20, 30, 40, and 50 mg/mL groups, respectively. MSs prepared at 20, 30, and 50 mg/mL displayed surface agglomeration, irregular clustering, and roughness, along with broad particle size distribution and higher SPAN. In contrast, MSs prepared with 40 mg/mL PEG-PLLA showed improved morphological uniformity. They also exhibited a narrower size distribution, with an average diameter of 61.507 ± 2.21 μm (*D*_10_ = 24.316 ± 1.42 μm, *D*_90_ = 117.991 ± 3.95 μm, SPAN = 1.523 ± 0.07) and the highest fraction of particles within the desired 30–60 μm range. Therefore, the 40 mg/mL formulation was selected for all further experiments.

#### 3.1.2. Concentration of PVA

**Figure 4:**
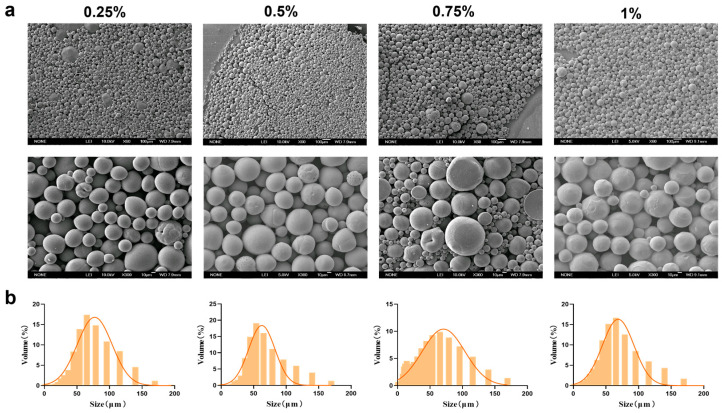
Characterization of MSs for PVA concentration optimization. (**a**) SEM micrographs of PEG-PLLA MSs and (**b**) Particle size distribution of PEG-PLLA MSs.

Based on the superior morphology and particle size distribution of the MSs, a PEG-PLLA concentration of 40 mg/mL was selected. Following this, we next optimized the concentration of PVA in the external aqueous phase (0.25%, 0.5%, 0.75%, and 1%) to assess its influence on MS formation. The corresponding results were presented in Figure 4 and SEM micrographs revealed that MSs were formed at all PVA concentrations tested. However, surface adhesion was observed at PVA concentrations of 0.75% and 1%. Particle size analysis showed that the volume proportion of MSs within the target range of 30–60 μm was 60%, 65%, 62%, and 58% for the 0.25%, 0.5%, 0.75%, and 1% PVA groups, respectively. Among these, MSs prepared with 0.5% PVA exhibited the highest proportion of particles in the desired size range. They also met the criteria of a relatively small average particle size (64.567 ± 2.34 μm) and a uniform distribution (SPAN = 1.68 ± 0.09; *D*_10_ = 16.682 ± 1.25 μm; *D*_90_ = 125.155 ± 4.12 μm). Therefore, 0.5% was selected as the optimal PVA concentration for the external aqueous phase.

#### 3.1.3. The Speed of Magnetic Stirring

**Figure 5:**
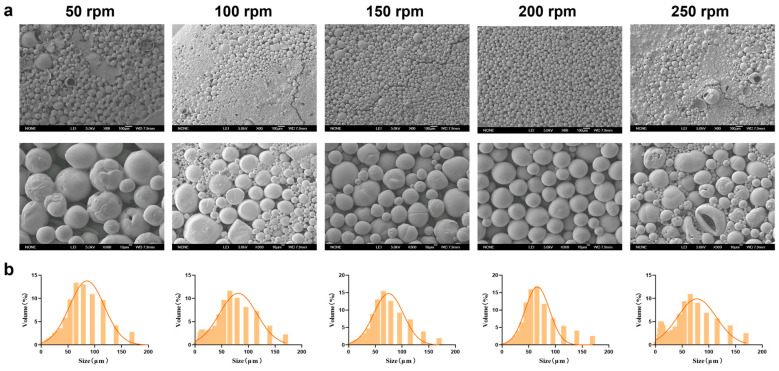
Characterization of MSs with optimized magnetic stirring speed. (**a**) SEM micrographs of PEG-PLLA MSs and (**b**) Particle size distribution of PEG-PLLA MSs.

Magnetic stirring speed significantly affects the physicochemical properties of MSs during preparation. After investigating the optimal PEG-PLLA concentration, we systematically evaluated how different stirring speeds (50, 100, 150, 200, and 250 rpm) influenced the physicochemical properties of the MSs. Based on the SEM results (Figure 5a), as the magnetic stirring speed increased, the physicochemical properties of MSs gradually improved. However, high shear forces caused droplet breakup and extensive fragmentation at 250 rpm. Particle size distribution showed that the volume fraction within 30–60 μm were 65%, 60%, 68%, 70%, and 55% for 50, 100, 150, 200, and 250 rpm, respectively (Figure 5b). Experimental results indicated that increasing the stirring speed generally reduced particle size and improved MS morphology. Nonetheless, excessively high speeds caused overshearing, which disrupted the newly formed droplets and reduced MS uniformity. MSs prepared at 200 rpm exhibited optimal morphology, a small average size (59.324 ± 2.15 μm), and a narrow distribution (SPAN = 1.586 ± 0.08; *D*_10_ = 18.972 ± 1.32 μm; *D*_90_ = 113.06 ± 3.87 μm). This condition also yielded the highest proportion of particles in the target size range. Therefore, 200 rpm was selected for subsequent experiments.

### 3.2. The Reproducibility of PEG-PLLA MSs

**Figure 6:**
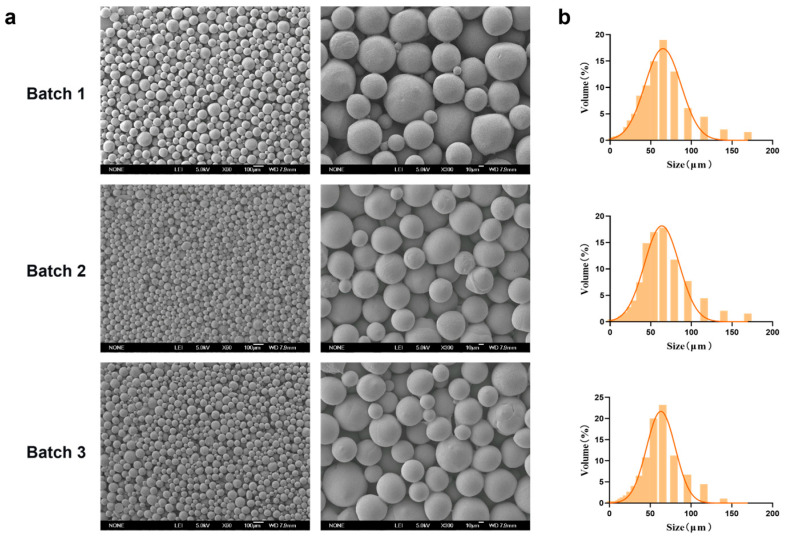
Characterization of MSs reproduced under optimal process parameters. (**a**) SEM micrographs of three batches of PEG-PLLA MSs and (**b**) Particle size distribution of each batch of PEG-PLLA MSs.

Representative SEM micrographs of the PEG-PLLA MSs prepared under the optimized conditions (PEG-PLLA concentration: 40 mg/mL; PVA concentration: 0.5%; stirring speed: 200 rpm) were presented in Figure 6. The MSs exhibited a uniform spherical morphology with smooth surfaces. Particle size distribution analysis showed that the volume percentage of MSs within the target range (30–60 μm) was 70%, 72%, and 71% across three independent batches, which indicated consistent batch-to-batch reproducibility. The average particle diameter was 58.98 ± 0.74 μm, with *D*_10_ = 20.786 ± 0.58 μm and *D*_90_ = 101.414 ± 2.15 μm. The SPAN was 1.37 ± 0.02, reflecting a narrow size distribution that met the typical particle size specifications for injectable facial fillers.

### 3.3. Effect of PEG-PLLA MSs on L929 Cell Viability

**Figure 7:**
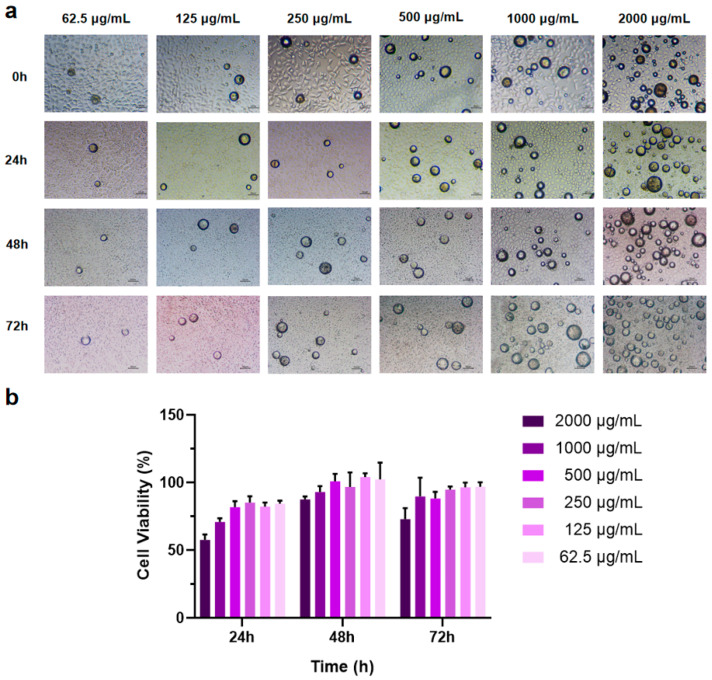
MSs of different concentrations were co-cultured with cells for 72 h. (**a**) Light micrographs of cells co-incubated with MSs. (**b**) Cell viability after co-incubation with MSs for 24 h, 48 h, and 72 h.

The CCK-8 assay was employed to assess the effect of PEG-PLLA MS treatment on cell viability. Sterile MS powder was added to RPMI 1640 complete culture medium to create suspensions at concentrations of 62.5, 125, 250, 500, 1000, and 2000 μg/mL. Cells were co-incubated with MS suspensions of different concentrations for 24 h, 48 h, and 72 h, respectively. The results showed that as the amount of MSs increased, cell viability decreased (Figure 7b). After co-incubation of cells with MSs for 48 h, the cell viability at this time reached roughly 1.3-fold the level measured at 24 h, and the cells exhibited significant proliferation. After co-culturing cells with MSs for 72 h, some groups showed a slight reduction in cell viability, whereas no significant inhibitory effects on cell proliferation were observed. These results indicated that PEG-PLLA MSs possess excellent biocompatibility. Simultaneously, the experimental results determined the optimal drug concentration, showing that PEG-PLLA MS suspension concentrations below 500 μg/mL have minimal impact on cell viability. Consequently, a suspension concentration of 500 μg/mL was chosen for subsequent cellular assays.

### 3.4. Screening of Optimal H_2_O_2_ Concentration for Modeling Cellular Senescence

**Figure 8:**
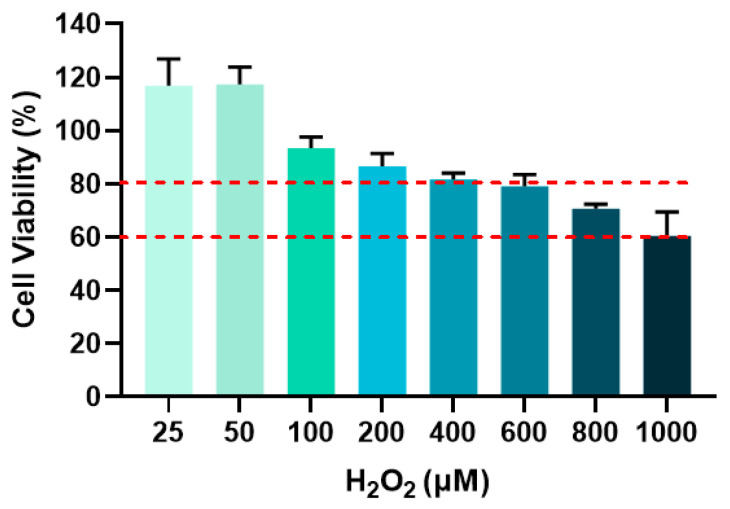
Cell viability after different concentrations of H_2_O_2_ treatment for 24 h.

The optimal H_2_O_2_ concentration for subsequent senescence modeling was determined by selecting groups showing cell viability between 60% and 80% [16]. Figure 8 displayed the cell viability in response to varying H_2_O_2_ concentrations and the red dashed lines defined the 60–80% viability range. As shown in Figure 8, cell viability decreased with increasing H_2_O_2_ concentration. At 800 μM H_2_O_2_, cell viability reached 70.64%, which falls within the target range. Therefore, 800 μM H_2_O_2_ was chosen for all subsequent cellular senescence experiments.

### 3.5. The Effect of PEG-PLLA MSs on the Content of Col-I and Col-III

**Figure 9:**
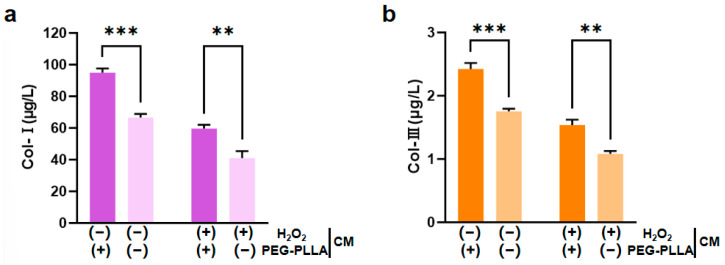
PEG-PLLA MSs enhanced collagen secretion in fibroblasts. (**a**) Col-I secretion level. (**b**) Col-III secretion level. The experimental groups are denoted using symbols where (+) indicates the presence and (−) indicates the absence of a treatment factor. The four groups are: Control [H_2_O_2_ (−), MSs (−)], MSs only [H_2_O_2_ (−), MSs (+)], H_2_O_2_ only [H_2_O_2_ (+), MSs (−)], and H_2_O_2_ + MSs [H_2_O_2_ (+), MSs (+)]. Data are mean ± SD (*n* = 3). ** *p* < 0.01; *** *p* < 0.001 vs. control.

L929 fibroblasts are key functional cells responsible for synthesizing and secreting ECM components. Under stimulation by relevant molecular signals, these cells can further regulate the expression of collagen genes, including types I and III; this regulatory effect is crucial for imparting mechanical strength and elasticity to the ECM [17,18]. Therefore, this study employed H_2_O_2_ to induce oxidative damage in L929 cells. H_2_O_2_ exposure triggered a substantial increase in intracellular reactive oxygen species (ROS), leading to cellular oxidative stress. The secretion levels of both Col-I and Col-III were significantly lower in H_2_O_2_-treated cells compared to untreated controls. This confirmed that oxidative impairment reduced the collagen-secreting capacity of the cells. Treatment with PEG-PLLA MSs increased Col-I and Col-III secretion in both the normal group and the oxidatively stressed group. In normal cells, the increase was approximately 1.5-fold (*p* < 0.001). In the cells under oxidative stress, levels increased approximately 1.45-fold (*p* < 0.01). This indicated that PEG-PLLA MSs can enhance collagen production not only under normal conditions but also in a state of oxidative injury (Figure 9). Notably, the stimulatory effect of PEG-PLLA MSs on collagen regeneration was more pronounced in normal cells than in oxidative stress-treated cells (*p* < 0.001). These results indicated that the PEG-PLLA MSs not only enhance collagen secretion in normal fibroblasts but also restore the collagen synthesis function of cells under oxidative stress.

### 3.6. The Effect of PEG-PLLA MSs on Hyaluronic Acid (HA) Content

**Figure 10:**
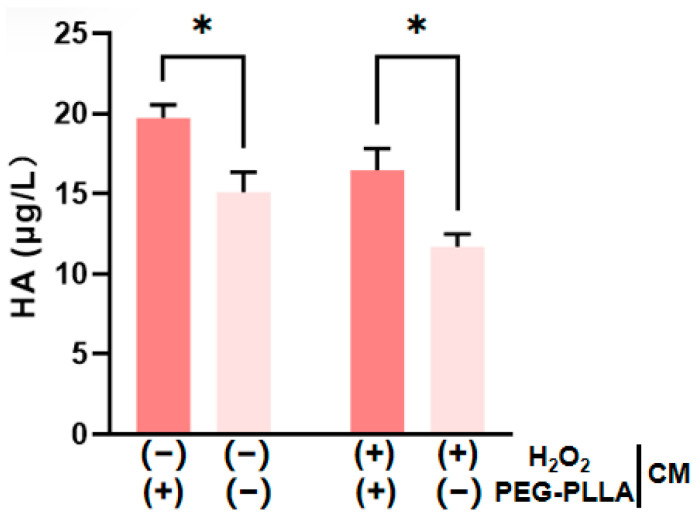
PEG-PLLA MSs enhance HA secretion by fibroblasts. The experimental groups are denoted using symbols where (+) indicates the presence and (−) indicates the absence of a treatment factor. The four groups are: Control [H_2_O_2_ (−), MSs (−)], MSs only [H_2_O_2_ (−), MSs (+)], H_2_O_2_ only [H_2_O_2_ (+), MSs (−)], and H_2_O_2_ + MSs [H_2_O_2_ (+), MSs (+)]. Data are mean ± SD (*n* = 3). * *p* < 0.05 vs. control.

HA is present in both the epidermal and dermal layers of the skin. Within the dermis, HA plays a key role in maintaining skin hydration and elasticity [19,20]. As shown in Figure 10, H_2_O_2_-induced oxidative stress significantly reduced HA secretion by cells compared to untreated controls. This confirmed that oxidative damage impairs HA production. PEG-PLLA MS treatment was subsequently applied to both normal and oxidatively stressed groups. The secretion levels were increased approximately 1.33-fold in the normal group and 1.42-fold in the oxidatively stressed group. The treatment significantly elevated HA secretion in both cellular groups (*p* < 0.05). The data demonstrated that PEG-PLLA MSs can enhance HA secretion in normal cells and rescue the impaired HA production in cells under oxidative stress.

### 3.7. The Antioxidant Effect of PEG-PLLA MSs

**Figure 11:**
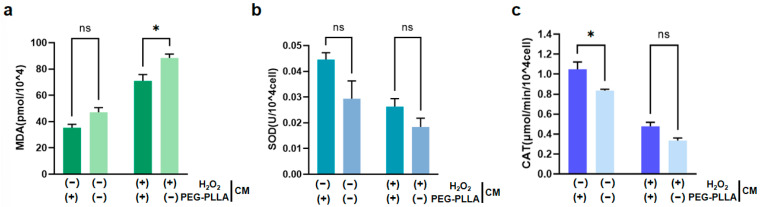
Effects of PEG-PLLA MSs on oxidative stress in fibroblasts. (**a**) MDA content, (**b**) SOD activity, and (**c**) CAT activity. The experimental groups are denoted using symbols where (+) indicates the presence and (−) indicates the absence of a treatment factor. The four groups are: Control [H_2_O_2_ (−), MSs (−)], MSs only [H_2_O_2_ (−), MSs (+)], H_2_O_2_ only [H_2_O_2_ (+), MSs (−)], and H_2_O_2_ + MSs [H_2_O_2_ (+), MSs (+)]. Data are mean ± SD (*n* = 3). ns, not significant; * *p* < 0.05 vs. control.

As shown in Figure 11a, treatment with H_2_O_2_ significantly increased MDA content in fibroblasts, indicating oxidative damage. Correspondingly, the activities of SOD and CAT were significantly reduced in H_2_O_2_-treated cells (Figure 11b,c). This suggested that the cellular antioxidant defense system was compromised under oxidative stress. Notably, PEG-PLLA MSs alleviated the H_2_O_2_-induced increase in MDA content in both normal and oxidatively stressed groups. In the normal group, MDA was reduced 1.5-fold, while a 1.56-fold reduction was observed in the oxidatively stressed group (*p* < 0.05). The MSs also modulated antioxidant enzyme activities. In normal cells, SOD activity increased 1.51-fold (not statistically significant) and CAT activity increased 1.35-fold (*p* < 0.05). Although increases in SOD (1.43-fold) and CAT (1.28-fold) activity were observed in cells under oxidative stress, the differences were not statistically significant. Therefore, PEG-PLLA MSs increased SOD and CAT activity in both cell groups. However, this elevation reached statistical significance solely in normal group, not in the oxidatively stressed group. These results demonstrated that PEG-PLLA MSs alleviated oxidative stress by mitigating damage and enhancing cellular antioxidant capacity.

## 4. Discussion

Polylactic acid (PLA) is a prominent synthetic, bio-based polyester. Owing to its biocompatibility and ability to undergo hydrolytic degradation into harmless metabolites, it presents considerable promise for a wide range of medical applications [21,22,23]. In 1995, the Food and Drug Administration (FDA) approved PLA and its derivatives as biodegradable materials for clinical use. This regulatory milestone paved the way for the development of numerous PLA-based biomedical products. These products now include absorbable sutures, anti-adhesion membranes, and guided tissue regeneration membranes [24,25]. However, the development of PLA is constrained by several factors. Its low crystallinity, high crystallization temperature, and slow crystallization rate severely limit its performance and applicability [26,27]. Modifying PLA is a promising strategy to address these issues. Effective methods include adding nucleating agents such as thermoplastic polyurethane (TPU) [28], PEG [29], and polyethylene oxide (PEO) [30]. Incorporating chiral structures, as in poly-L-lactic acid (PLLA), is another successful approach. The FDA first approved PLLA in 2004 for the treatment of HIV-associated facial lipoatrophy [31]. Then, in 2009, the approval was extended to include the improvement of age-related deep wrinkles in the general population [32]. Since then, PLLA has been widely used as a soft tissue filler. One representative product, Sculptra, consists of PLLA MSs suspended in a sodium carboxymethylcellulose gel. After injection, these MSs induce a local tissue response that recruits macrophages and fibroblasts. This cellular interaction promotes collagen synthesis, resulting in smoother skin and improved texture [33]. In practice, PLLA can be applied to superficial skin layers, deep tissues, and bone contouring regions. It is commonly used to correct wrinkles, nasolabial folds, and other facial contour defects. Despite being recognized as a biocompatible material, there have been reports of significant inflammatory reactions and other adverse events associated with its use. These reports highlight the need for careful evaluation of its clinical safety [34,35]. To address this, we selected PEG-PLLA as the raw material. The introduced PEG/mPEG chains increase crystallinity and crystallization rate while lowering the crystallization temperature and improving hydrophilicity. Moreover, they slow the release of L-lactic acid during degradation [36]. Therefore, MSs from PEG-PLLA retain the collagen-stimulating properties of PLLA while potentially reducing its inflammatory responses [37].

Beyond material properties, the size and size distribution of MSs are key determinants of injectable efficacy. An optimal size must be large enough to prevent passage through capillary walls and phagocytosis by dermal macrophages, yet not so large as to cause needle clogging [38]. Li reported that MSs with a diameter of 20–75 μm evade phagocytosis by THP-1 cells for at least 24 h, thus mitigating the risk of acute inflammation associated with macrophage uptake. Moreover, subcutaneous injection of MSs with a diameter within this range in Sprague–Dawley rats showed no chronic adverse reactions, such as foreign body granulomas or inflammatory nodules, in H&E-stained sections. Therefore, to ensure safety and injectability, the particle size of the MSs in this study was controlled within 30–60 μm [39]. Common techniques for MS preparation include emulsion solvent evaporation, phase separation, spray drying, and SPG membrane emulsification. SPG membrane emulsification is a technique that enables the creation of micro-sized emulsions and particles with a narrow size distribution through uniformly sized membrane pores [40,41,42,43]. This method has the advantages of simple operation, uniform particle size, and good stability. In this study, PEG-PLLA MSs with a uniform particle size distribution and an average diameter of 30–60 μm were successfully fabricated using SPG membrane emulsification. Furthermore, a single-factor optimization approach was systematically employed to optimize three key process parameters: oil phase concentration, aqueous phase concentration, and magnetic stirring speed. During the optimization of MS preparation, the oil phase concentration was initially investigated. PEG-PLLA concentration critically governed the MSs’ physicochemical properties (e.g., morphology and size). When only the oil phase concentration was varied, an increase in PEG-PLLA concentration led to larger droplet sizes. This is because higher viscosity under the same pressure reduces droplet breakup efficiency, thereby resulting in the formation of larger emulsion droplets from the dispersed phase [44]. Lower concentrations produced porous, irregular MSs, whereas higher concentrations yielded smoother, denser MSs with concomitantly larger particle sizes [45]. Subsequently, the concentration of PVA in the aqueous phase was optimized. The PVA concentration influences the surface tension, density, and viscosity of the aqueous phase, all of which are critical for emulsion stability. An increase in PVA concentration raises the viscosity of the continuous phase, which slows down its flow rate and reduces the shear force acting on the emerging droplets. As a result, droplet detachment from the membrane surface is delayed, leading to larger droplet sizes and broader size distribution [46]. Finally, the magnetic stirring speed was systematically investigated. During membrane emulsification, droplet formation was governed by the shear force generated by the stirring of the continuous phase [47]. This shear force acts on droplets forming at the membrane pores, detaching them into the continuous phase. Therefore, the stirring speed is a key parameter affecting MSs’ quality: lower speeds result in weaker shear forces, slower droplet detachment, and larger particle sizes; conversely, higher speeds produce smaller droplets [48]. However, excessively high speeds caused overshearing, which disrupted the droplets. To confirm the reproducibility of the optimized process, three independent batches of MSs were prepared under identical optimal conditions. The batches exhibited consistent and favorable physicochemical properties with minimal inter-batch variation. These results confirmed the establishment of a stable and reproducible membrane emulsification process for PEG-PLLA MSs, thereby achieving the primary objective of this study.

The PEG-PLLA MSs exhibited no significant cytotoxicity. At concentrations below 500 μg/mL, cell viability was maintained or even enhanced compared to the control. Notably, viability at 48 h was consistently higher than at 24 h across all groups. This time-dependent improvement may be explained by two interrelated factors: an initial cellular adaptation period to the new material, leading to transiently restrained growth at 24 h, followed by acclimation and more robust proliferation by 48 h [49,50]. Additionally, the gradual release of trace components from the MSs might provide subtle signals that further support cell cycle progression [51]. Although, high concentrations induced a transient suppression of cellular activity, the cells demonstrated adaptive recovery. These findings collectively corroborate the excellent biocompatibility of the PEG-PLLA MSs.

We hypothesize that the anti-aging effects of the MSs are mediated by two primary mechanisms: the direct stimulation of key ECM components, including Col-I, Col-III, and HA; and the mitigation of cellular oxidative stress induced by excess ROS. It is anticipated that by reducing oxidative stress, the MSs will decrease MMP-mediated ECM degradation, thereby promoting overall ECM homeostasis [52,53]. ROS, including the superoxide anion (O_2_•^−^), H_2_O_2_, and the hydroxyl radical (•HO), directly damage cellular macromolecule-proteins, lipids, and nucleic acids. Such cumulative damage can result in cellular dysfunction, growth arrest, and ultimately, cell death [54]. MDA is a principal end product of lipid peroxidation; consequently, its level serves as a direct biomarker for lipid peroxidation and an indirect indicator of oxidative stress severity. In the cellular enzymatic antioxidant system, SOD and CAT play central roles. In addition, SOD scavenges O_2_•^−^ by catalyzing its dismutation to H_2_O_2_ and O_2_. CAT then detoxifies H_2_O_2_ by converting it to H_2_O and O_2_, thereby preventing its conversion into the highly destructive •OH via the Fenton reaction [55]. Together, SOD and CAT maintain redox homeostasis and protect against oxidative damage. Therefore, the efficacy of the MSs against oxidative stress was comprehensively evaluated through the concurrent measurement of MDA, SOD and CAT levels. In addition, the accumulation of ROS in the skin contributes to skin aging by degrading key ECM components, such as collagen, elastin, and hyaluronic acid. This degradation leads to a loss of skin elasticity, the formation of wrinkles, and increased laxity [56]. Of these, Col-I is the principal fibrous component responsible for the structural integrity, stability, and tensile strength of connective tissues [57,58,59]. It constitutes over 90% of the body’s total collagen and serves as the major structural protein in skin (representing 80–85% of the dermal extracellular matrix), bone, tendon, ligament, cornea, and visceral organs, including the liver, lungs, and heart [60,61]. Col-III forms delicate, elastic reticular fibers that are essential for the proper assembly and stabilization of collagen fibrils [62]. It accounts for approximately 5–20% of total body collagen, making it the second most abundant collagen subtype after Col-I [63]. HA interacts with collagen and elastin fibers in the extracellular matrix, contributing to the skin’s mechanical elasticity [60,64,65]. Due to its unique physicochemical structure, HA possesses a remarkable capacity to bind and retain water, thereby preserving dermal moisture. Consequently, reduced dermal HA contributes to characteristic signs of skin aging, including wrinkles, roughness, and sagging [66]. Excessive ROS promotes inflammation and activation of transcription factors that drive degradation of the skin extracellular matrix. Subsequently, both Col-I and Col-III are cleaved by endopeptidases of the matrix metalloproteinase (MMP) family [67,68]. Consequently, the density of Col-I and Col-III in skin declines with advancing age [69]. Similarly to collagen, the reduction in HA in aged skin is not solely caused by decreased synthesis. MMPs produced by senescent skin cells actively degrade HA, along with other ECM components such as proteoglycans and glycosaminoglycans [70]. Based on the proposed dual mechanism, we conducted in vitro validation using normal and oxidative stress-treated fibroblasts. Therefore, we measured the production of Col-I, Col-III, and HA alongside oxidative stress parameters. Treatment with the MSs increased the secretion of all three ECM components in both cell states. Critically, in stressed cells, it also significantly decreased MDA levels and boosted CAT and SOD activities. This provides direct experimental support for the hypothesis that MSs promote ECM synthesis and mitigate oxidative damage.

A limitation of this study is the absence of a direct comparison with pure PLLA MSs, precluding the ability to definitively attribute the enhanced biocompatibility and performance to the PEG modification. Future studies incorporating this control are needed to isolate the specific role of PEG. In addition, future research should build upon these cellular findings by translating them into two key directions. First, in vivo studies in appropriate animal models are necessary to evaluate the safety, biodegradability, and long-term efficacy of the MSs, thereby providing definitive evidence for functional tissue regeneration. Second, the molecular mechanisms should be elucidated, focusing on analyzing relevant fibroblast signaling pathways to delineate the link between attenuated oxidative stress and elevated levels of ECM components, thus validating the proposed hypothesis.

## 5. Conclusions

This study successfully developed uniformly sized PEG-PLLA MSs via SPG membrane emulsification and confirmed their biosafety through cytotoxicity evaluation. In addition, the results of in vitro evaluation experiments indicate that co-culture with PEG-PLLA MSs markedly enhanced the expression of Col-I, Col-III, and HA in normal cells and restored their expression in cells under oxidative stress. The activities of key antioxidant enzymes were also enhanced in both conditions. These initial results suggested that PEG-PLLA MSs are capable of promoting ECM component production and mitigating oxidative stress in cells. By establishing optimized and reproducible processing parameters, this work introduced a reliable fabrication strategy for PEG-PLLA MSs with controlled particle size, offering a promising platform for the development of novel injectable fillers.

## Data Availability

The datasets used and analyzed during the current study are available from the corresponding author upon request.
